# 3D fractures analysis and conservation assessment of wrought iron javelin through advanced non-invasive techniques

**DOI:** 10.1038/s41598-023-37179-w

**Published:** 2023-06-22

**Authors:** Martina Bernabale, Flavio Cognigni, Chiara Mancini, Anacleto Proietti, Francesco Mura, Daria Montanari, Lorenzo Nigro, Marco Rossi, Caterina De Vito

**Affiliations:** 1 Department of Earth Sciences, Sapienza University of Rome, P.le Aldo Moro 5, 00185 Rome, Italy; 2 Department of Basic and Applied Sciences for Engineering (SBAI), Sapienza University of Rome, Via Antonio Scarpa 14, 00161 Rome, Italy; 3 Department Italian Institute of Oriental Studies - ISO, Sapienza University of Rome, Circonvallazione Tiburtina 4, 00185 Rome, Italy

**Keywords:** Microscopy, Scanning electron microscopy, Materials science, Techniques and instrumentation, Imaging techniques

## Abstract

3D imaging is a powerful tool of high resolution and non-destructive imaging technology for the study of ancient weapons and military technology, which reveals the original microstructures and corrosion patterns that threaten these artefacts. Here we report quantitative analysis of the 3D distribution and the orientation of fractures, and uncorroded metal particles within a wrought iron javelin unearthed at the Phoenician-Punic site of Motya, Italy. The study aimed to gain a better understanding of the relationship between corrosion and local stresses within the artifact and to evaluate its manufacturing technology, as well as the effects of post-treatment with Paraloid B72 on concretion and mineralized layers. The cracks were quantified in terms of content, size, and orientation. The condition of artefact storage was evaluated by a multi-analytical approach, including X-ray microscopy, field emission electron microscopy and micro-Raman spectroscopy. The results indicated that a specific technique was used to create a sturdy, lightweight javelin with a central shaft for piercing or thrusting. The fractures appear elongated in the direction of the longitudinal axis of the blade, showing the forging direction of the original metallic block. The study concluded that the artifact had not yet been stabilized due to the presence of lepidocrocite.

## Introduction

The stabilization of archaeological iron finds is an everlasting problem in the field of conservation. Several different treatment methods, materials and procedures have been used in the conservation practice over the years^1,2^. Modern 3D visualization, virtual reconstruction, modeling and data processing technologies are useful tools for controlling and evaluating degradation processes^3,4^ and restoration treatments^5^. The aim of this paper is to present the capabilities and potential of X-ray microscopy in a metallic artefact, which allows visualization and accurate tri-dimensional spatial analysis of the exterior and interior of an iron javelin.

Chemical composition, structure and corrosion products may hint at the origin, manufacturing technology and storage conditions of the object. The elemental analysis provides key information about the nature of raw materials for provenance studies and the role of alloying elements^6–9^, whereas structural information addresses questions of manufacturing techniques and corrosion processes^10,11^.

Research on the three-dimensional spatial arrangement and topological relationships of fracture networks and metals is crucially for systematic understanding of corrosion evolution in archaeological iron works. The defects left on the surfaces of artefacts results in non-uniform stress distribution inside the structures that might cause micro-cracks or pits and provide the main pathways for water and oxygen to reach deep within the artefacts^12^.

Recent surveys of fracture networks using X-ray computed tomography have been conducted in medical sciences^13^, construction industry^14,15^, energy materials^16^, environmental sciences^17^ and in tectonics to evaluate the fracture geometries of reservoirs and fracture propagation behavior in naturally fractured reservoirs^18,19^.

Here, we propose this approach for archaeological iron artefacts, aiming at (1) quantifying these fractures and (2) understanding topological relationships that affect the corrosion path. To better understand the relationship between the corrosion and growth of defects and the local stress, and phase changes that cause them, we need both imaging and spectroscopic analyses. Thus, we explore how this can be achieved by different techniques such as Field Emission Electron Microscopy (FESEM-EDS), X-ray Microscopy (XRM) and Micro-Raman Spectroscopy to study corrosion product layers in archaeological iron javelin to find the causes of the corrosion phenomena during their burial period and after excavation.

In terms of digitization, visualization, non-destruction, topologizing and quantitation, X-ray Microscopy is adopted to characterize corrosion models. XRM can provide morphological inner features, such as amount, distribution and shape of defects, porosity and slag inclusions, that are related to the manufacturing process (i.e. defects, slag inclusions, internal cracks). In addition, important information on the conservation status can be obtained from the determination of the mineralization phases and the corrosion products that can be easily mapped using Micro-Raman spectroscopy. This technique represents an excellent tool in the identification of the corrosion phases occurring at microscopic scale, providing a fingerprint of the compound under study in a non-destructive and non-invasive way^12,20^. In this study Micro- Raman system was also used to detect the existence of conservation treatments in the metal artefacts.

**Figure 1:**
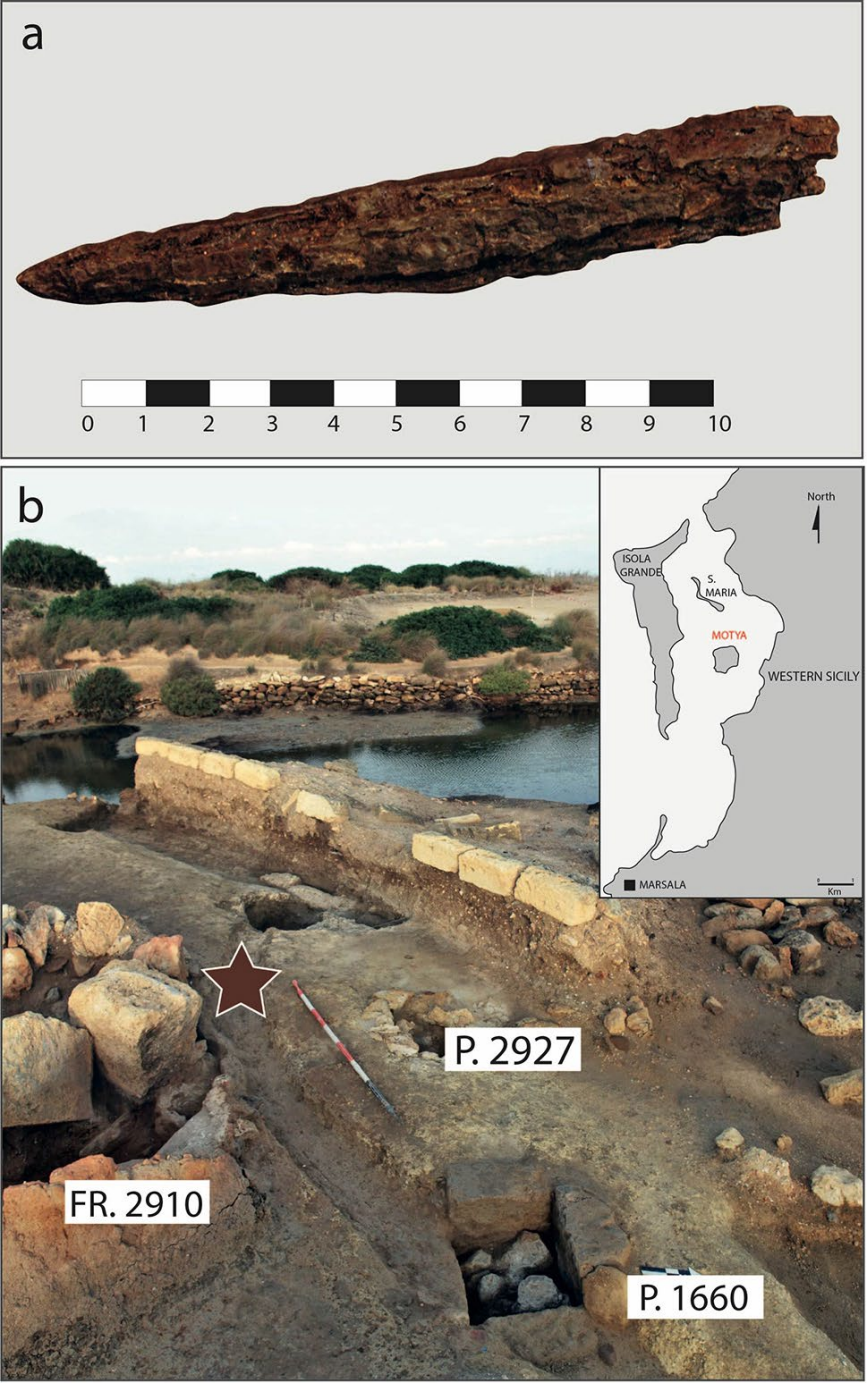
Photograph of the javelin under investigation in this study. (**a**) Macroscopic view of the javelin MC.09.191 and (**b**) photo of Area C of “Kothon” (Motya, Sicily) where the javelin was buried. The map has been created with Adobe Illustrator 27 (https://www.adobe.com/products/illustrator.html).

Javelin at issue (MC.09.191) of Fig. 1 has been found at the Punic site of Motya, across the harbour of modern-day Marsala on the western coast of Sicily (Italy). It was found in 2009 in the south-western side of Motya, in the Area C of “Kothon” outside the furnace F.2910, near the Well P.1660. The well, with a square mouth, was already in use at least from the end of the eighth century BC, but, after the middle of the sixth century BC, it was used for ritual purposes^21^. Javelin MC.09.191 has a shaft handle with two flaps with circular section, generally obtained by fusion in double matrix with inert body or by rolling the metal sheet around the pole. Sometimes javelins of this type have a bronze band with a parallel line decoration at the end of the shaft. This element, also found in some javelins from the necropolis of Palermo, reinforced the handle^22,23^.

After its discovery, the javelin was treated with acrylic resins such as Paraloid B72 (ethyl methacrylate methyl acrylate copolymer) in order to render back, as much as possible, cohesion, physical properties and mechanical strength to the artefacts and to produce a barrier layer that excludes moisture and oxygen from contact with the surface of the object. Solution of tannic acid in water was applied. Tannic acid, a complex organic acid that reacts with iron to form ferric tannate, helps to inhibit susceptible areas of the surface from reacting with water vapor.

The javelin is a medium-range and close-quarters weapon that has been used for centuries, particularly in ancient times as a part of military tactics and athletic competitions. It typically consists of a shaft made of wood or bamboo, with a pointed metal or wooden tip at one end and a grip or cusp at the other end. The javelin has good ballistic properties, allowing it to travel through the air in a stable and predictable manner, and a typical range of between 35 and 40 m, depending on the weight and design of the javelin. It can be thrown with great accuracy and force, making it an effective weapon for both hunting and warfare^24,25^.

Studying the manufacturing technique of the javelin can provide important clues about the military, technological, economic, and social aspects of ancient societies.

## Results and discussion

### Identification of corrosion products

**Figure 2:**
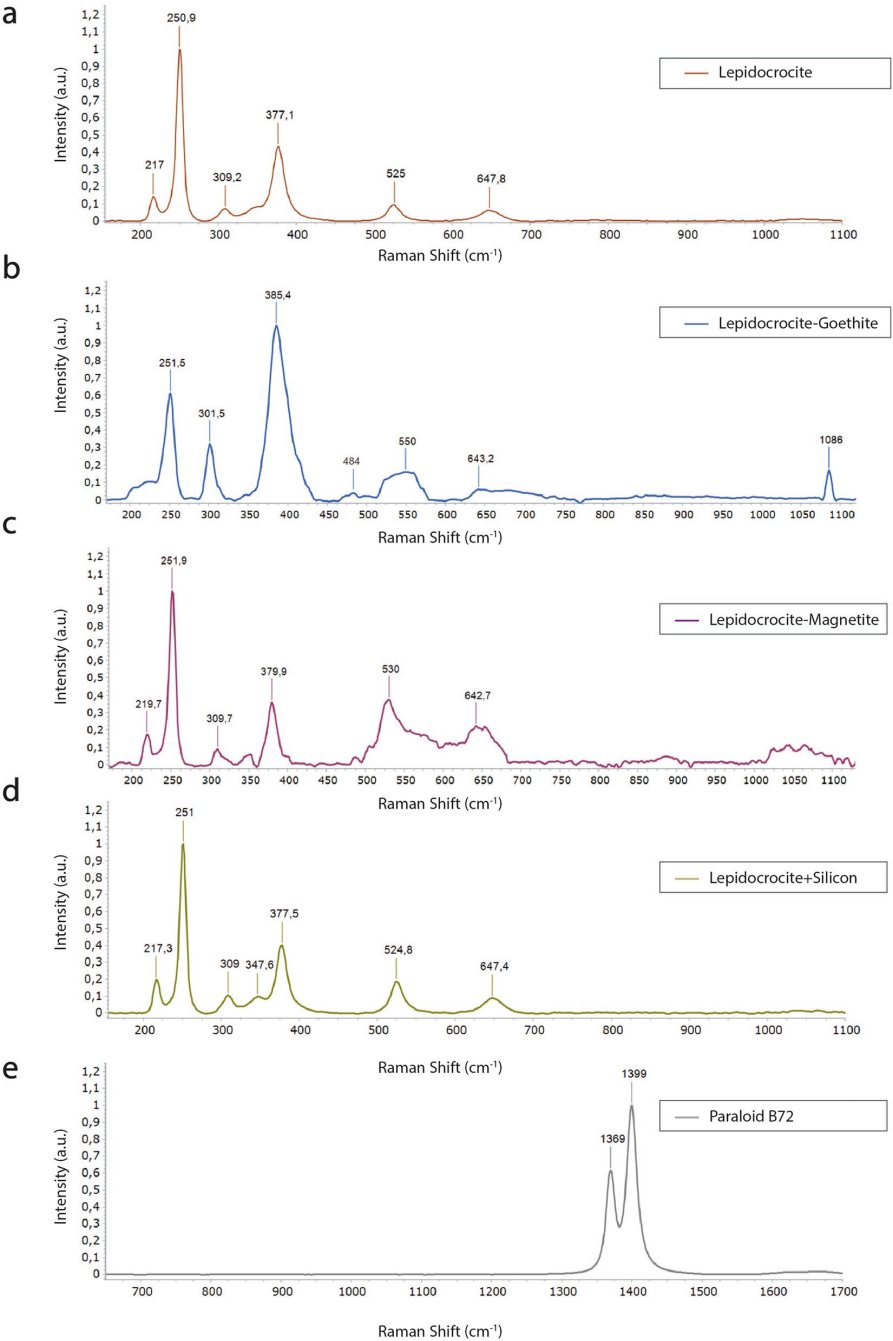
Mineralogical composition of javelin patina. Raman spectra of (**a**) lepidocrocite, (**b**) lepidocrocite and goethite, (**c**) lepidocrocite and magnetite, (**d**) lepidocrocite and silicon, (**e**) Paraloid B72.

Micro-Raman spectroscopy was used to explore the degree of alteration on rust scales and possible polymer degradation effects of the acrylic resin. By means of Raman spectroscopy, it was found that the surface of the javelin was formed mainly by lepidocrocite (γ‐FeO(OH)), Fig. 2). Lepidocrocite, identified through the bands 217, 250, 309, 377, 525, 647 cm^−1^ (Fig. 2a), is one of the most characteristic mineral phases of metal pieces that have suffered corrosion after being buried in the moist sediments and in the presence of dissolved oxygen^12,20,26–28^. Deviations among lepidocrocite spectra can be attributed to the wide range of crystallinity of these compounds. Locally on the same areas of the artefact, the strong peak at 385 cm^−1^ with small peak at 484 cm^−1^ indicates the presence of goethite (α-FeOOH) in mixture with lepidocrocite (γ-FeOOH) (Fig. 2b); in addition, the vibration at 1083 cm^−1^ is a possible match with calcite due to the symmetric stretching vibration of the carbonate groups.

The spectrum acquired from a crust extrusion revealed a more noticeable peak at 642 cm^−1^ could be related to magnetite (Fe_3_O_4_) (Fig. 2c).

The peak at 528 cm^−1^ attributed to lepidocrocite appears slightly shifted to lower wavenumbers (524 cm^−1^), due to the presence of silicon whose main peak occurs at 520.5 cm^−1^(Fig. 2d).

Moreover, the only bands related to the presence of the acrylic resin have been detected in Fig. 2e, for which the analyzed spectral range shift to higher wavenumbers (600–1700 cm^−1^). Indeed, in the Raman spectra of Paraloid B72, the CH_2_ deformation mode appears at around 1370 cm^−1^, while the CH_3_ stretching mode appears at around 1399 cm^−1^. These two modes are related to the vibrations of the carbon-hydrogen bonds in the ethyl methacrylate and methyl acrylate units, respectively^29^. The presence of only these two peaks is probably due to photo-oxidative stress of the co-polymer as extensively reported in the literature^30–34^. Indeed, several experiments have demonstrated that scission reactions prevail over cross-linking ones in Paraloid B72, containing solely ethyl and methyl esters, while resins which contain longer ester groups are more sensitive to fast and extensive cross-linking^30^.

### Fracture characterization method using X-ray microscopy

The cohesion of the original surface and the adhesion to the core metal, as well as the formation of cracks and quantification of non-corroded metal core, were studied using X-ray Microscopy^3,4^. In addition, by analyzing the distribution of fracture sizes, it is possible to obtain information about the strength and toughness of the material and evaluate the probability of crack propagation and the ultimate failure of the material.

Understanding the fracture characteristics of iron javelin is important not only for understanding the technological production but also for preservation and conservation of the artefact.

**Figure 3:**
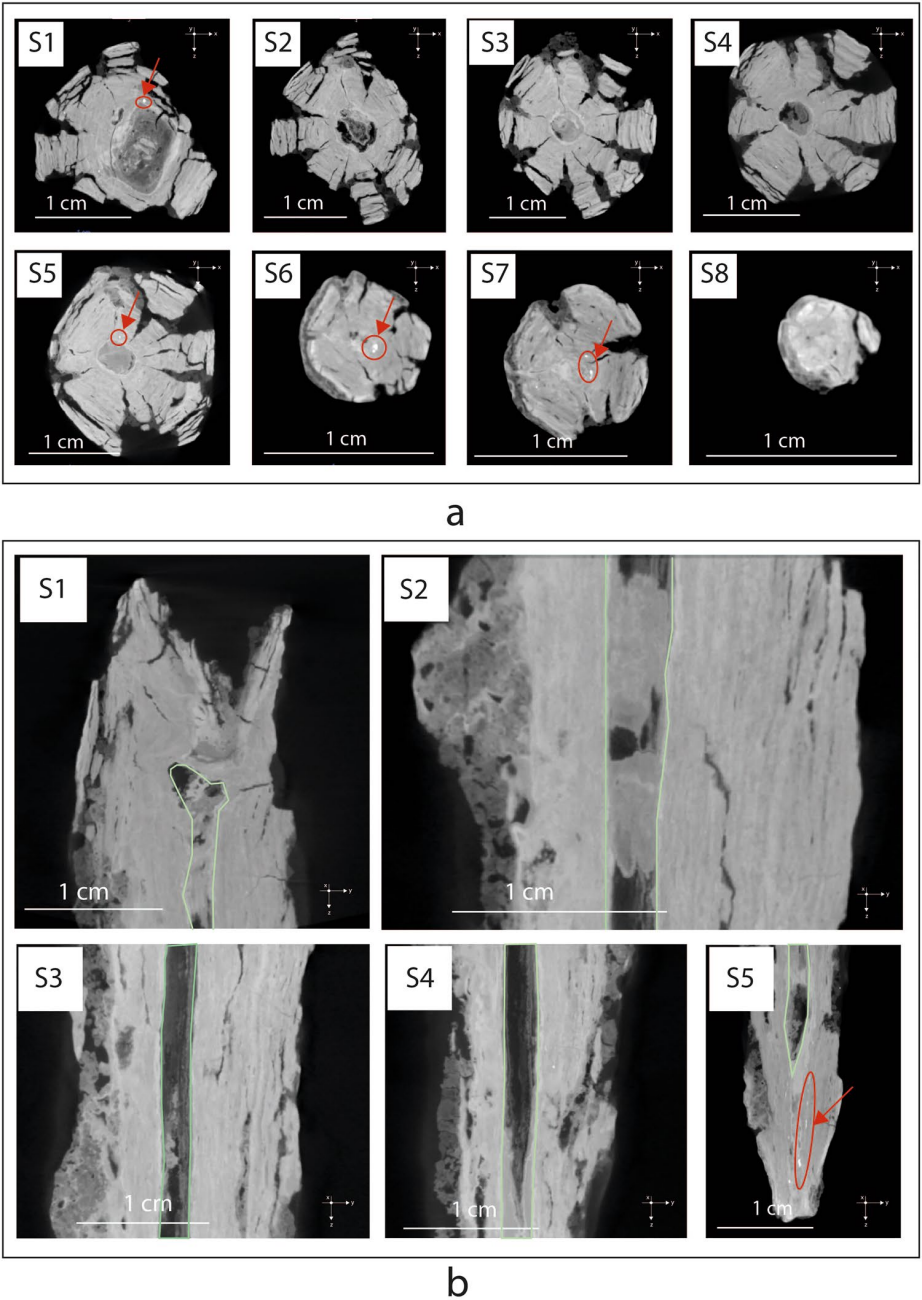
X-ray microscopy slices illustrating javelin microstructure. Virtual XZ cross-sections (**a**) and ZY cross-sections (**b**) of the 3D model of the javelin, showing numerous cracks having radial cross-section, uncorroded metal particles (in red circles) and a big fissure penetrating the entire section and parallel to its longitudinal axis. Light gray levels correspond to the uncorroded iron core and dark gray to the corrosion products, according to the absorption coefficient of each region of the artifact and to the length of the X-ray beam path inside the sample.

Figure 3a,b shows eight virtual XZ cross-sections and five ZY cross-sections of the 3D model of javelin. On XRM images, light gray levels correspond to the uncorroded iron core and dark gray to the corrosion products, according to the absorption coefficient of each region of the artifact and to the length of the X-ray beam path inside the sample. The sample shows a circular section and several cracks having radial cross-section, similar to the deterioration of logs.

A further phenomenon observed in the object consists of a big fissure (2, 5 mm) penetrating the entire section and parallel to its longitudinal axis, with a distribution resembling and often joined to the above-mentioned cracks. This structure could be due to a particular technique used in ancient times to manufacture weapons such as spears, javelins, and arrows. In this process, a long, narrow strip of wrought iron is cut and then flattened. This strip is then rolled around a rod to create a cone-shaped form, which is then hammered to create a hollow tube with a central shaft. The resulting tube is then cut to the desired length and shaped into a javelin.

This technique allowed for the production of strong, lightweight weapons with a central shaft that could be used for piercing or thrusting. This fissure is often filled with soil materials and inclusions of corrosion products, which act as natural consolidators and hinder the further embrittlement of the corrosion product layer^35^.

**Figure 4:**
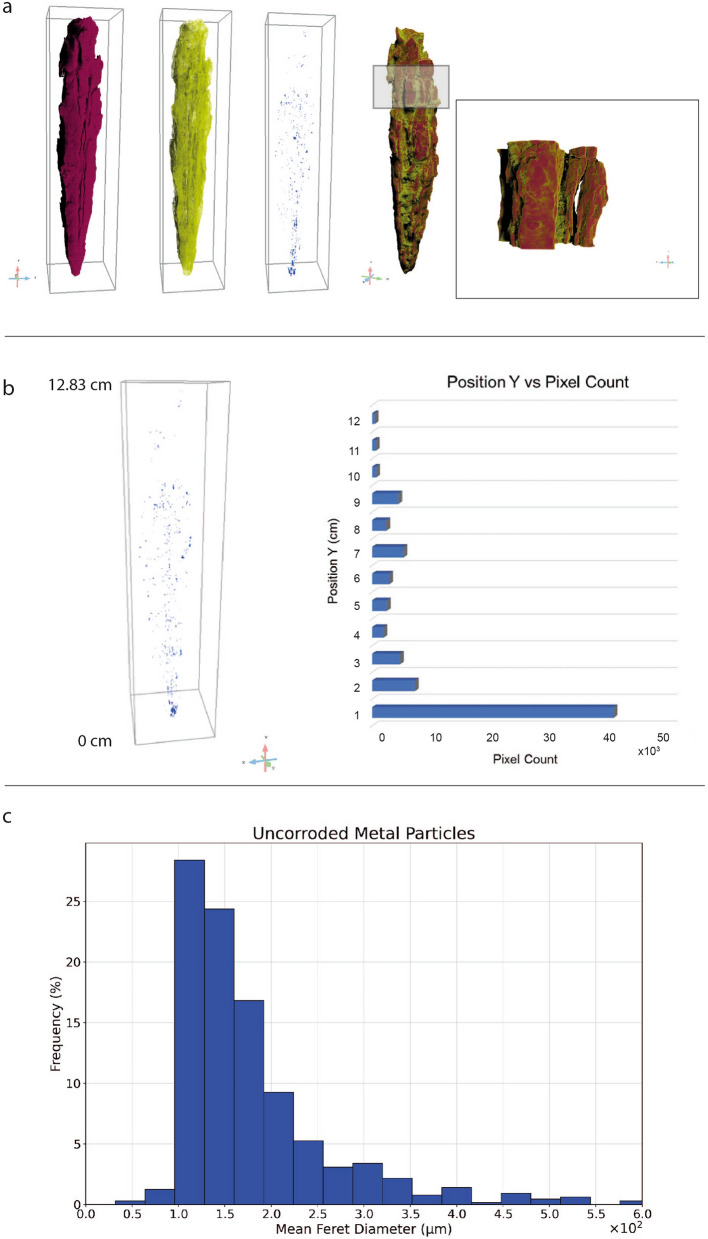
3D reconstruction of the javelin and quantification of uncorroded metal particles (**a**) 3D reconstruction of the javelin and segmentation of the different phases composing the sample: the corrosion layers (bordeaux), soil (yellow) and uncorroded metal particles (blue) on the left side. An enlarged view of the upper part of the javelin is highlighted by the box revealing the presence of a great quantity of exfoliations accompanied with discontinuous aggregates appearing on the surface. This figure has been created with Dragonfly Pro Version 2022.1 Built 1259 (https://theobjects.com/index.html) (**b**) The uncorroded metal particles are mainly concentrated in the first centimeter of the javelin (0 ÷ 1 cm), close to the tip, where a total number of 40,452 voxels representing this phase was registered. In the range 2 ÷ 12 cm the average number of pixels representing the uncorroded metal particles is 2956. The graph has been generated with Microsoft Excel (https://www.microsoft.com/it-it/microsoft-365/excel) (**c**) The mean Feret diameter histogram of the uncorroded metal particles reveals that more than 91% of uncorroded metal particles were smaller than 0.3 mm, while the largest one was approximately 0, 6 mm in diameter. The graph has been created with Google Colab (https://colab.research.google.com/?hl=it).

The detection and segmentation of several phases composing the javelin, hence the corrosion products (bordeaux), soil (yellow) and uncorroded metal particles (blue) is reported in Fig. 4a. The process of voxels labeling, i.e*.* segmentation, is carried out on a XRM scan with a voxel size of 31.4 μm. Therefore, every detail smaller than three voxels, as rule of thumb, is excluded from the following discussion, since it is considered to be noise.

It can be seen that a soil layer (yellow) covered the surface of the sample and significant corrosion layers (bordeaux) were formed by conversion of iron to rust. As shown in detail in the XRM detail of a selected region of interest (ROI) of the javelin, there are a great quantity of exfoliations accompanied with discontinuous aggregates appearing on the surface. As it progresses getting closer to the tip of the javelin, the debris and flaky corrosion products on the surface of the corrosion layer gradually decreased. The percentage volume of each phase resulted by imaging segmentation was calculated considering also the fractures. In this case, the uncorroded metal occupies only 0.01% of the total volume of the object, while about 75.76% has been transformed into rust and 11.77% is soil. The calculated volume fraction of crack was 11.74%. Note that these phases are also visible in the radiographs of Fig. 3a,b.

Observing the three-dimensional distribution of uncorroded metal particles (blue), reported in Fig. 4b (left), it was highlighted a significant concentration of this phase in correspondence of the tip of the javelin. Using the *basic measurements* tool in Dragonfly Pro (Version 2022.1 Built 1259) it was computed the center of mass along the Y-axis and the voxel count of every uncorroded metal particle in the Multi-ROI. This information was correlated to obtain the plot of Fig. 4b (right). In the first centimeter of the javelin (0 ÷ 1 cm) a total number of 40,452 voxels was registered, representing the uncorroded metal particles presence. Moving away from the tip of the nail an apparent decrease of uncorroded metal particles, in terms of voxel count, can be appreciated. In the range 2 ÷ 12 cm the average number of pixels representing the uncorroded metal particles is 2956. The quantitative evaluation of the mean Feret diameter histogram of Fig. 4c indicates that more than 91% of uncorroded metal particles were smaller than 0.3 mm, while the largest one was approximately 0,6 mm in diameter.

**Figure 5:**
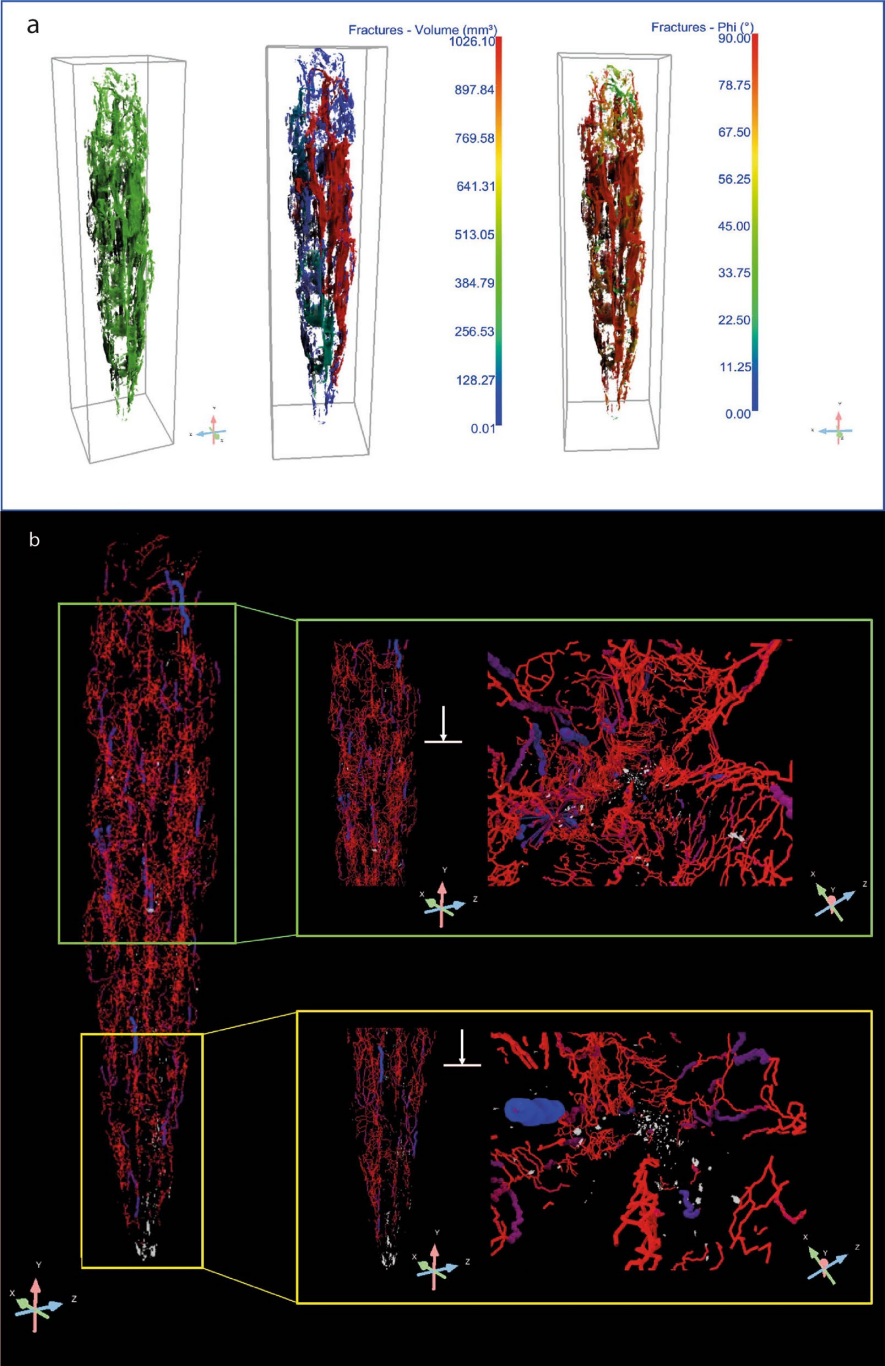
Fractures characterization by X-ray Microscopy (**a**) Segmented fractures with angle inclination (phi), (**b**) 3D skeletonization showing the spatial distribution and the length of fractures and their connectivity. This figure has been created with Dragonfly Pro Version 2022.1 Built 1259 (https://theobjects.com/index.html).

Once the fractures have been segmented and reported in green [see Fig. 5a (left)], each crack is treated as an individual object that can be characterized via *basic measurement*s tools available for 3D Multi-ROIs. For instance, the volume of the fractures was obtained by computing the volume occupied by the labeled voxels representing each fracture. The results are reported in a colorimetric 2D/3D scale map in Fig. 5a (middle) revealing the presence of a larger crack in red (1026.10 mm^3^) running through the entire length of the javelin with a preferential distribution on the right side, close to the surface. Then, the orientation of fractures, described by the angle 0° < ϕ (Phi) < 90°, is given by the mutual orientation of the longest axis of an object (i.e. crack) and the Z-axis, as depicted in Fig. 5a (right). The cracks show a predominantly longitudinal orientation regarding the loading direction parallel to the Z-axis, i.e. ϕ = 90°, which is a result of the production technique of the object, which has been forged with two-by-two opposing and orthogonal hammering. Thus, the cracks are oriented in the direction of the length of the element. Due to the stress generated by multiple forging operations in more directions, the core of the artifact is the most fragile and less compact.

A further step of the investigation required the understanding of the relationship between the three-dimensional distribution of fractures and the presence of uncorroded metal particles over the entire volume of the javelin. With this purpose, the ROI representing the fractures was used to create a *dense graph* which consists in a 3D model of connected areas, i.e. fractures, in which the spheres and lines representing those areas and their connections can be examined in a 3D view with respect to the uncorroded metal particles (white), as shown in Fig. 5b and Supplementary Movie 1. The creation of the *dense graph* allowed us to demonstrate that the presence of uncorroded metal particles coincides with the absence of fractures, as confirmed by the detailed view from the green and yellow box in Fig. 5b (left). Observing the distribution of fractures with respect to the uncorroded metal particles along the Y-axis of the javelin (right) it is evident that no fractures reach the metal particles. This phenomenon can be explained as it follows. When metal corrodes, it forms iron oxide (rust), which can flake off originating cracks and fractures over its volume. These cracks allow oxygen and moisture from the atmosphere to penetrate deeper into the metal, accelerating the corrosion process. Considering the long exposure of the javelin to corrosion agents, humidity and moisture from the atmosphere almost the entirety of the metal volume was corroded. However, some uncorroded metal particles are still present where the absence of fractures is registered witnessing its preservation. These considerations provide a clear explanation to the significant presence of metal particles in the first centimeter of the javelin (see Fig. 4b) since this is the region that shows the minimum number of fractures.

### Chemical composition and morphology of the coating

**Figure 6:**
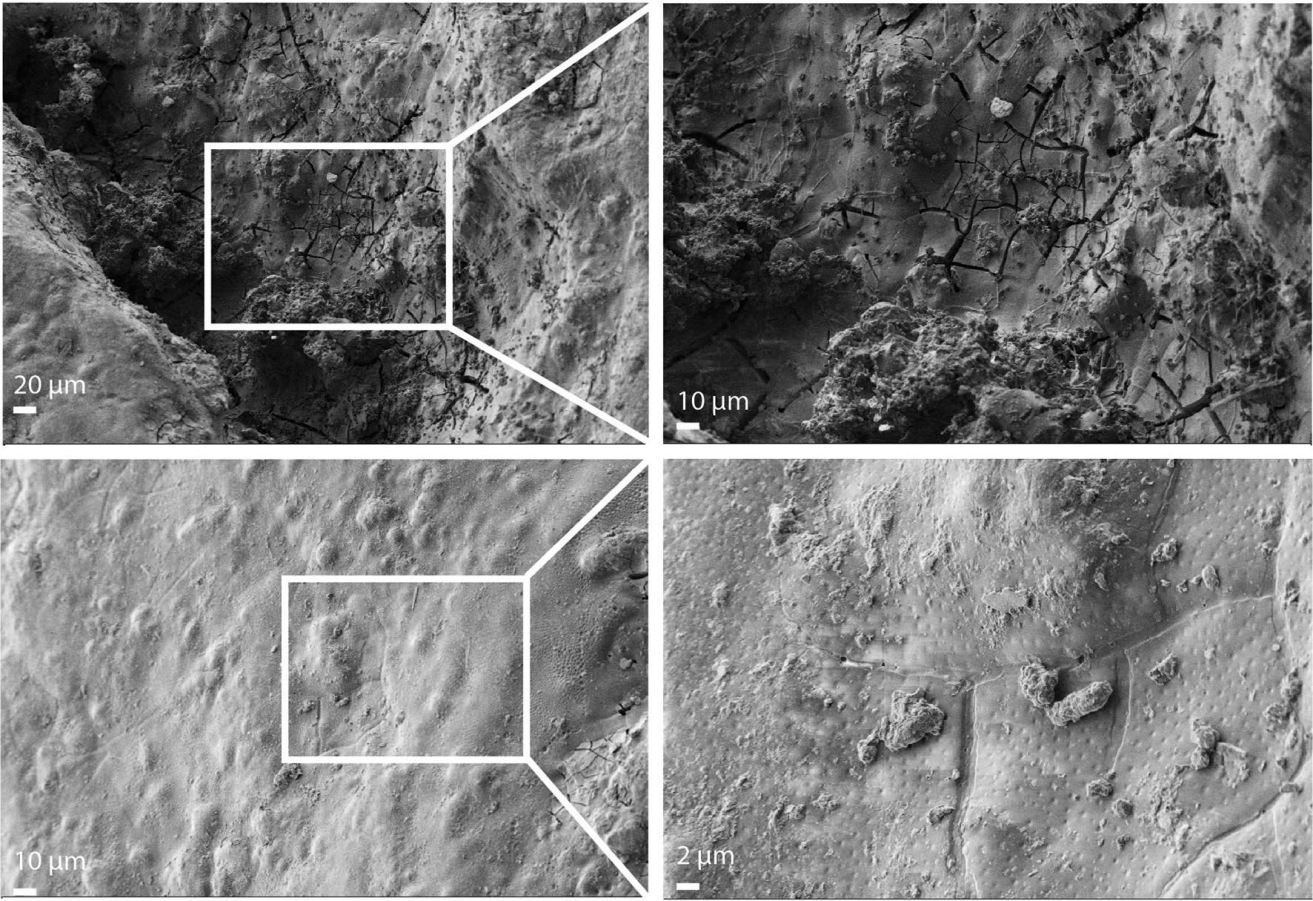
Surface characterization by FESEM. High resolution FESEM images of the javelin, showing surface topography of the artefact.

High resolution images of the treated sample surface were obtained by field-emission scanning electron microscopy (FESEM). The corrosion products cover the entire surface of the sample and have a grain-like shape. Figure 6 shows the topography of the zone on the iron sample surface after Paraloid B72 coating application. Several cracks and voids on its surface can be observed. In addition, the enlarged view of the surface shows many pits. Consequently, the porous structure and the existence of crevices in the corrosion layer create channels that allow protective resin to penetrate. On the tip of the artefact, there are almost no cavities on the surface of the corrosion layer, the surface is uniform, less porous and covered with a complete protecting coating.

### Assessment of potential of XRM for archaeometallurgy and CH microstructural monitoring

This paper texted the recent technological developments in geology and engineering fields for cultural heritage applications, aiming to assess the potential of XRM for the in-situ preservation of artefacts in Museum and Art gallery. We use a metal artefact of artistic and historical interest as a model to demonstrate how insights from recent advances in 3D X-ray imaging can help to understand the structure and properties of such artefacts.

In particular, X-Ray Microscopy allowed the identification of structural components and morphological features that can be related to its manufacturing process, life cycle, and conservation state of the artefact. We assessed the depth, location, direction and arrangement of the cracks visible from the surface and to notice the presence of inner and hidden uncorroded metal particles in the same *dense graph*. We showed that the presence of uncorroded metal particles coincides with the absence of fractures, confirming that structural breaks influence the durability and the performance of metal artefacts as well as the corrosion depth propagation. Tracking the fracture network configuration has also given important information about the manufacturing of the object such as thermal and mechanical treatments. Indeed, the presence of a big fissure penetrating the entire object suggests that the javelin was rolled around the pole to create a strong, lightweight weapon with a central shaft that could be used for piercing or thrusting. Therefore, we suggest that 3D modeling coupled with X-ray Microscopy should become a fundamental practice for the inventory of historical objects in order to generate high resolution and high-quality images and to document archaeological objects and perform structural health monitoring analysis for preservation. The future work will involve the development of a novel strategy for monitoring cultural heritage artefacts damage in corrosive environments over time to explore the relation between the location of metal particles and the fractures on the object’s surface.

Another good practice consists in monitoring by Micro-Raman spectroscopy the corrosion products and, once applied, the protective Paraloid B72 film, in order to observe the variations in the corrosion stabilization and polymerization of the resin. Indeed, as the exposure time increases and the rust layers become thicker, the active lepidocrocite could be partially transformed into inactive goethite and a stratified dual rust-layer structure formed by a porous outer lepidocrocite layer and a more compact inner goethite layer may become consolidated.

## Methods

### Corrosion products and coating characterization

Micro-Raman analyses were performed at room temperature with the confocal inVia™ Raman Spectrometer (Renishaw) with 250 mm focal length and a 50 × short working-distance NPLAN objective (NA = 0.75, Leica Microsystems). The excitation line employed is a HeNe continuous-wave diode-pumped solid-state laser (Renishaw) with λ = 632.81 nm. The signal has been acquired in the 150–1350 cm^−1^ spectral range with an exposition time between 1 and 5 s and a laser power usage from 0.2 to 10 *mW*, according to the compound under analysis. In order to get a clear spectrum, 10 accumulations have been acquired for each measure. The signal is dispersed by a holographic grating of 1800 l/mm and collected by a Peltier-cooled CCD detector. All the post-processing activities (normalization, smoothing, peak label) have been carried out on the software WiRETM 4.4; normalized intensities of each band result from the ratio of their height and the one of the most intense peaks of the spectrum.

### Microstructure characterization

FESEM-EDS investigations were carried out by Zeiss Auriga 405 microscope at Sapienza Nanoscience & Nanotechnology Laboratories (SNN-Lab) of the Research Center on Nanotechnology Applied to Engineering (CNIS) of Sapienza University.

### Cracks quantification

X-ray microscopy (XRM): was performed using a laboratory X-ray microscope (Zeiss, Xradia Versa 610) available at the Research Center on Nanotechnology Applied to Engineering (CNIS) of Sapienza University of Rome which is part of the open infrastructure for Advanced Tomography and Microscopies (ATOM). Since the javelin dimensions exceed the maximum Field Of View (FOV) that can be reached in a normal scan mode, the object was scanned in the automatic vertical stitch mode. The object was divided in six sub-volumes, each of which was scanned setting the sample-to-detector distance to 92.7 mm, and source-to- sample distance was 78 mm. The voltage and power of the X-ray beam were set to 150 kV and 23 W. Scans were performed from 0° to 360° using a 0.4 × objective, the exposure time for each projection was set to 1 s and 1601 projections were acquired. A total scanning time of circa 7 h was required for all the sub-volumes. Acquired images were obtained with a pixel size of 31.4 μm and binned (2 × 2 × 2). The stack of projection images was reconstructed automatically using the Reconstructor module of Zeiss Scout and Scan control software (Version 16.1.13038.43550) via Feldkamp-Davis-Kress algorithm.

Image processing: At the end of the reconstruction process a TIFF stack was imported in Dragonfly Pro software (Version 2021.1 Build 1259, Object Research System) for post processing.

A detailed description of XRM working principles and components can be found in^36^.

## Supplementary Information

Supplementary Video 1.

Supplementary Legends.

## Data Availability

All data generated or analysed during this study are included in this published article [and its supplementary information files].
