# Supplementary material for: 3D fractures analysis and conservation assessment of wrought iron javelin through advanced non-invasive techniques
Source: Sci Rep. 2023 Jun 22;13:10142. doi: 10.1038/s41598-023-37179-w (PMC10287633; doi:10.1038/s41598-023-37179-w)
Supplement: Supplementary file 2 — Supplementary Legends. [file 41598_2023_37179_MOESM2_ESM.pdf]

3D fractures analysis and conservation assessment of wrought iron javelin through advanced non-invasive techniques

Martina Bernabale[1], Flavio Cognigni[2], Chiara Mancini[2], Anacleto Proietti[2], Francesco Mura[2], Daria Montanari[3], Lorenzo Nigro[3], Marco Rossi[2] and Caterina De Vito[1]

Supplementary movie legend:

Movie S1: 3D graph of fractures to investigate their spatial arrangement with respect to uncorroded metal particles.
